# A New Oleanolic Acid Derivative against CCl_4_-Induced Hepatic Fibrosis in Rats

**DOI:** 10.3390/ijms18030553

**Published:** 2017-03-06

**Authors:** Hongjun Xiang, Yaotian Han, Yuzhong Zhang, Wenqiang Yan, Bing Xu, Fuhao Chu, Tianxin Xie, Menglu Jia, Mengmeng Yan, Rui Zhao, Penglong Wang, Haimin Lei

**Affiliations:** 1 School of Chinese Pharmacy, Beijing University of Chinese Medicine, Beijing 100102, China; iloveygr@163.com (H.X.); han_yaotian@bucm.edu.cn (Y.H.); ywq3226925@163.com (W.Y.); weichenxubing@126.com (B.X.); chufhao@163.com (F.C.); xietx1993@163.com (T.X.); jml15001290933@126.com (M.J.); yanmengmeng@bucm.edu.cn (M.Y.); zr1012@bucm.edu.cn (R.Z.); 2 Department of Pathology, Beijing University of Chinese Medicine, Beijing 100102, China; zyz100102@126.com

**Keywords:** oleanolic acid derivative, carbon tetrachloride-induced, liver fibrosis, histological study, acute toxic test, pharmacokinetic

## Abstract

A novel hepatoprotective oleanolic acid derivative, 3-oxours-oleana-9(11), 12-dien-28-oic acid (Oxy-Di-OA), has been reported. In previous studies, we found that Oxy-Di-OA presented the anti-HBV (Hepatitis B Virus) activity (IC_50_ = 3.13 µg/mL). Remarkably, it is superior to lamivudine in the inhibition of the rebound of the viral replication rate. Furthermore, Oxy-Di-OA showed good performance of anti-HBV activity in vivo. Some studies showed that liver fibrosis may affiliate with HBV gene mutations. In addition, the anti-hepatic fibrosis activity of Oxy-Di-OA has not been studied. Therefore, we evaluated the protective effect of Oxy-Di-OA against carbon tetrachloride (CCl_4_)-induced liver injury in rats. Daily intraperitoneally administration of Oxy-Di-OA prevented the development of CCl_4_-induced liver fibrosis, which was evidenced by histological study and immunohistochemical analysis. The entire experimental protocol lasted nine weeks. Oxy-Di-OA significantly suppressed the increases of plasma aspartate aminotransferase (AST) and alanine aminotransferase (ALT) levels (*p* < 0.05). Furthermore, Oxy-Di-OA could prevent expression of transforming growth factor β1 (TGF-β1). It is worth noting that the high-dose group Oxy-Di-OA is superior to bifendate in elevating hepatic function. Compared to the model group, Oxy-Di-OA in the high-dose group and low-dose group can significantly reduce the liver and spleen indices (*p* < 0.05). The acute toxicity test showed that LD_50_ and a 95% confidence interval (CIs) value of Oxy-Di-OA were 714.83 mg/kg and 639.73–798.73 mg/kg via intraperitoneal injection in mice, respectively. The LD_50_ value of Oxy-Di-OA exceeded 2000 mg/kg via gavage in mice. In addition, a simple and rapid high performance liquid chromatography-ultraviolet (HPLC-UV) method was developed and validated to study the pharmacokinetic characteristics of the compound. After single-dose oral administration, time to reach peak concentration of Oxy-Di-OA (C_max_ = 8.18 ± 0.66 μg/mL) was 10 ± 2.19 h; the elimination half-life and area under the concentration-time curve from *t* = 0 to the last time of Oxy-Di-OA was 2.19 h and 90.21 μg·h/mL, respectively.

## 1. Introduction

Liver fibrosis is a reversible wound-healing response to acute or chronic hepatocellular injury [1]. Hepatic fibrosis and cirrhosis are major health problems that disturb millions of people worldwide. The high morbidity associated with hepatic fibrosis underlines the need for novel preventive and therapeutic approaches [2,3,4]. Therefore, it is necessary to design effective, specific, and safe novel preventive and therapeutic approaches for liver fibrosis.

**Figure 1:**
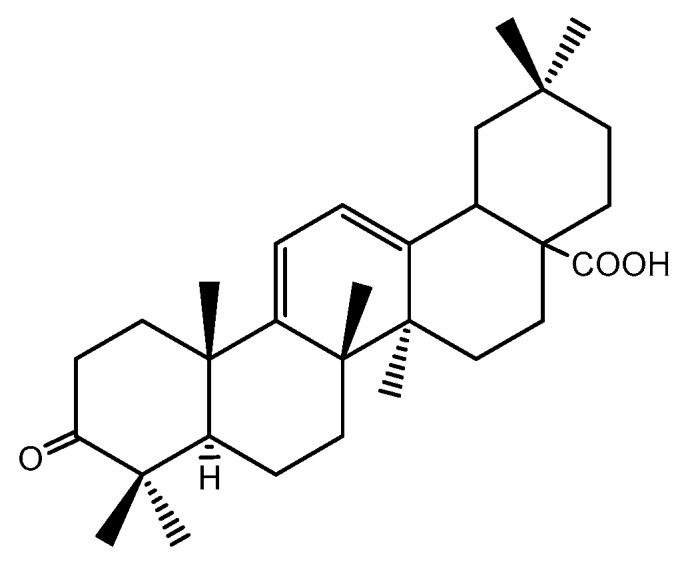
Structure of 3-oxours-oleana-9(11), 12-dien-28-oic acid (Oxy-Di-OA).

Oleanolic acid, an important active ingredient originated from *Ligustrum lucidum*, has been widely used for hepatoprotection in the clinic [5,6,7,8]. However, the low bioavailability has restricted its wider application [9]. Thus, it is necessary to design and synthesize a better derivative of oleanolic acid by structure modification. In previous studies, we synthesized 12-dien-28-oic acid (Oxy-Di-OA, Figure 1) and evaluated its anti-HBV activity. It can not only inhibit the secretion of HBsAg (Hepatitis B surface Antigen) (inhibition rate, 90.52% ± 1.78%) but also decrease the secretion of HBeAg (Hepatitis B e-Antigen) (reduction rate, 42.31% ± 3.53%). Additionally, it was superior to the positive drug (Lamivudine) in the inhibition of the rebound of the viral replication rate [10]. Host immune response and HBV gene mutations may influence the development and severity of liver fibrosis. In addition, liver fibrosis is the natural wound-healing process of necroinflammation caused by chronic HBV infection [11,12]. Thus, we decided to investigate the anti-fibrosis activity of Oxy-Di-OA. Progressive accumulation of the fibrillar extracellular matrix (ECM), including collagen produced mainly by hepatic stellate cells (HSCs) through the process termed activation in the liver, is the consequence of reiterated liver tissue damage due to infective (mostly hepatitis B and C viruses) or toxic/drug-induced processes, etc. The process may result in clinically evident hepatic fibrosis and liver cirrhosis [13]. However, rats and mice are immured to the infection of hepatitis B and C viruses [14,15]. Consequently, the present study was designed to investigate the anti-fibrosis effect of Oxy-Di-OA in rats and a series of experiments were conducted.

In this paper, we intended to evaluate the anti-hepatic fibrosis activity of Oxy-Di-OA in the case of CCl_4_-induced liver fibrosis in rats. Presently, histological assessment can provide direct information on the hepatoprotective effect of Oxy-Di-OA in CCl_4_-induced hepatic fibrosis in rats. Hepatotoxicity was biochemically assessed by serum aspartate aminotransferase (AST) and alanine aminotransferase (ALT). There are some studies showing that TGF-β1 has a close relationship with liver fibrosis. TGF-β1 is one of the most important cytokines leading to liver fibrosis and is closest to the development and progression of liver fibrosis and extracellular matrix (ECM) metabolism [16,17]. Thus, Immunohistochemical staining of the indicator were used to evaluate the activity of Oxy-Di-OA against CCl_4_-induced liver fibrosis in rats. Liver and spleen indices are also important indicators to detect the effect of Oxy-Di-OA in CCl_4_-induced liver fibrosis in rats. Moreover, an acute toxicity test was also used to evaluate the toxicity effect of Oxy-Di-OA in rats. In addition, a simple and rapid HPLC-UV method was developed to study the pharmacokinetic characteristics of Oxy-Di-OA.

## 2. Results

### 2.1. Histological Assessment

**Figure 2:**
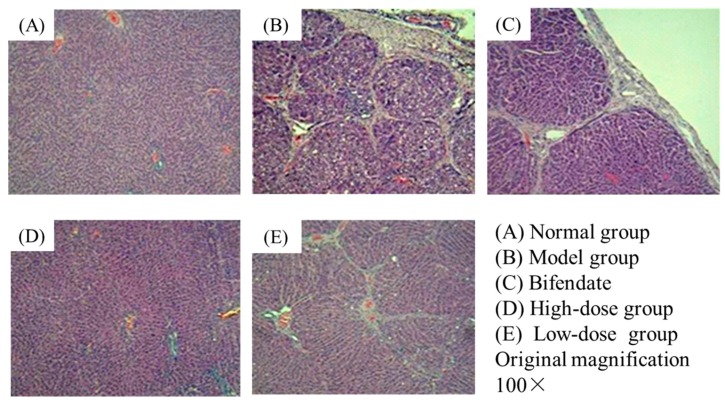
Histological examination of rat livers stained with hematoxylin-eosin (H&E). (**A**) Normal group, without any abnormal morphological alternations; (**B**) model group, showing marked morphological disruption; (**C**) bifendate (10.5 mg/kg), showing notable alleviation in abnormal areas; (**D**) high-dose group (Oxy-Di-OA, 28 mg/kg), showing marked regression and alleviation in the abnormal areas; and (**E**) low-dose group (Oxy-Di-OA, 14 mg/kg), showing moderate regression of morphological changes.

Histological evaluations provided direct assessment of the protective effects of Oxy-Di-OA on CCl_4_-induced liver fibrosis. The rats’ liver tissues of the normal group showed no apparent pathological changes in hepatic lobular architecture and hepatocytes (Figure 2A). However, in the model group, severe pathological alternations appeared, including interstitial fibrosis, centrilobular necrosis, considerable fatty degeneration of the hepatocytes, and massive collagen fibers in the portal and central areas (Figure 2B). The groups treated with Oxy-Di-OA with low and high doses appeared to relieve the pathological injuries to different degrees compared with the model group, respectively. The low-dose group displayed a moderately reduced severity of hyperplasia in liver tissue and liver fibrosis (Figure 2E). Moreover, compared with the model group, the high-dose group and the bifendate demonstrated obvious alleviation in abnormal areas and hepatocytes returned to a healthy state (Figure 2C,D). Especially in the high-dose group, the phenomena of fibrosis, fatty degeneration, and other necrosis are not observed.

The pictures of histological assessment are shown in Figure 2.

### 2.2. Immunohistochemical Staining

**Figure 3:**
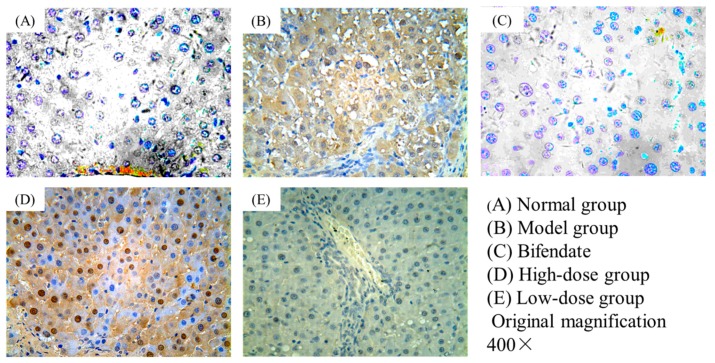
Immunohistochemical staining showed the expression of transforming growth factor β1 (TGF-β1). (**A**) Normal group, showed no expression of TGF-β1; (**B**) Model group, showed a high expression of TGF-β1; (**C**) Bifendate (10.5 mg/kg), showed a low expression of TGF-β1; (**D**) High-dose group (Oxy-Di-OA, 28 mg/kg), showed a lower expression of TGF-β1 compared to model group; (**E**) Low-dose group (Oxy-Di-OA, 14 mg/kg), showed a lower expression of TGF-β1 compared to model group.

The liver tissues in the rats of the normal group showed no expression of TGF-β1 (Figure 3A). It is obvious that the expression of TGF-β1 was very high in the model group (Figure 3B). However, compared to the model group, the high-dose (Figure 3D) and low-dose group (Figure 3E) displayed a visible lower expression. At the same time, the bifendate group showed a low expression of TGF-β1 (Figure 3C). In general, the obtained results revealed that Oxy-Di-OA could prevent expression of TGF-β1, and exert its anti-hepatic fibrosis activity.

The pictures of immunohistochemical staining are displayed in Figure 3.

### 2.3. Effects of Oxy-Di-OA on Serum Biochemistry

Table 1 lists the serum levels of ALT and AST, two sensitive indicators of liver damage and hepatic function. The serum activities of both ALT and AST were significantly increased in the model group compared with those in the normal group. However, compared with the model group, Oxy-Di-OA at two different doses significantly prevented the CCl_4_-induced increase of serum activity of ALT and AST.

### 2.4. The Effects of Oxy-Di-OA on Liver and Spleen Indices

The results showed that the liver and spleen indices in the model group were significantly higher than the normal group (3.72% ± 0.50% and 0.28% ± 0.09% vs. 2.70% ± 0.18% and 0.17% ± 0.03%, *p* < 0.01), indicating that CCl_4_ caused serious toxic effects in rats. However, administration with Oxy-Di-OA at high doses, these indices were significantly lower than the model group (3.15% ± 0.36% and 0.26% ± 0.05% vs. 2.70% ± 0.18% and 0.17% ± 0.03%, *p* < 0.05, Table 2), indicating that Oxy-Di-OA at high doses appears to be beneficial in CCl_4_-induced liver injury and inhibited the development of liver fibrosis in rats.

The available data of liver and spleen indices are listed in Table 2.

### 2.5. Acute Toxicity Test In Vivo

#### 2.5.1. Acute Toxicity Assay via Intraperitoneal Injection

The summary of the Oxy-Di-OA acute toxicity assay is expressed as the value of LD_50_ and 95% confidence interval (CIs), as shown in Table 3. According to the results in Table 3, the LD_50_ and 95% CIs of Oxy-Di-OA were calculated as 714.83 mg/kg and 639.73–798.73 mg/kg by Käber assessment, respectively. The acute toxicity data indicates that 600 mg/kg of Oxy-Di-OA was harmless for further in vivo study in mice. One hour after the experiment, symptoms such as slow movement and a decrease in aggressiveness were observed at high dose.

#### 2.5.2. Acute Toxicity Assay via Gavage

The LD_50_ value exceeded 2000 mg/kg by oral administration of Oxy-Di-OA.

### 2.6. Pharmacokinetic Study

#### 2.6.1. Method Validation

##### Specificity

**Figure 4:**
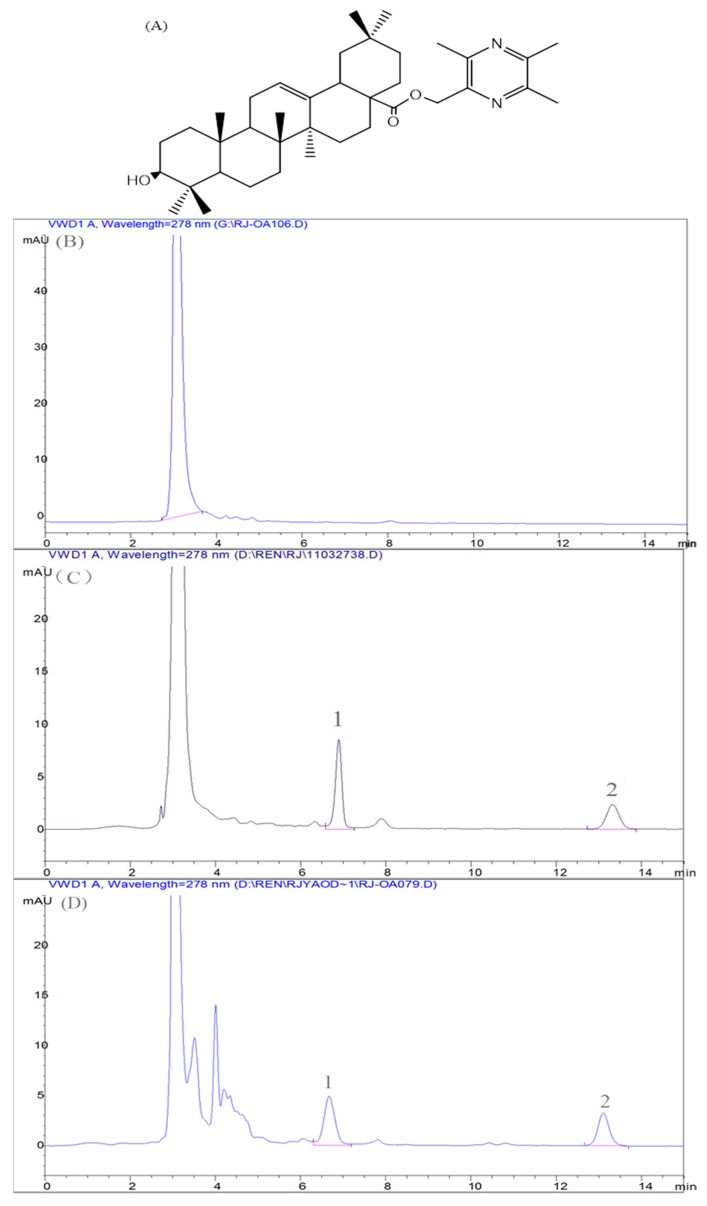
Representative high performance liquid chromatography (HPLC) chromatograms of the analytes detected at 278 nm. (**A**) Structures of internal standard (IS); (**B**) blank rat plasma; (**C**) blank rat plasma spiked with Oxy-Di-OA and IS; and (**D**) blank rat plasma sample collected at 12 h after administration of Oxy-Di-OA (500 mg/kg) added with IS; 1 Oxy-Di-OA; 2 IS.

Typical chromatograms of Oxy-Di-OA in rat plasma are presented in Figure 4. The satisfactory peak shape and resolution between peaks were achieved under the chromatographic conditions. Furthermore, no other endogenous substances strongly interfered with the determination of Oxy-Di-OA. The retention time for Oxy-Di-OA and internal standard (IS) was approximately at 6.9 and 13.2 min, respectively.

##### Calibration Curve and Linearity

The calibration curve exhibited good linearity over a range of 0.255–20.40 μg/mL in rat plasma.

The equation of linear regression was *Y* = 0.1769*X* + 0.046. *X* represented the ratio of the Oxy-Di-OA peak area over the IS area, and *Y* was the concentration of Oxy-Di-OA in rat plasma. The linear correlation coefficient (*r*) was 0.9993.

##### Precision, Limit of Detection, and Quantitation

The intra- and inter-day precisions are displayed in Table 4. RSD means the relative standard deviation. The limit of quantitation was 0.255 μg/mL and the limit of detection was 0.191 μg/mL. The intra- and inter-day precision was determined as values of RSD. It ranged from 0.54% to 2.43% and 1.76% to 4.27%, respectively. Hence, the present method for quantitative Oxy-Di-OA in biosamples was reliable and reproducible.

##### Extraction Recovery and Stability

The relative extraction recovery and absolute extraction recovery of Oxy-Di-OA in rat plasma are shown in Table 5. *C*_o_ means the observed concentration and *C*_t_ represents the theoretical concentration. *A*_o_ is the mean peak area (*n* = 5) obtained by the quality control (QC) samples after extraction, and *A*_t_ is the mean peak area (*n* = 5) generated by the working solutions of the same concentration as the QC samples.

The average relative recoveries of Oxy-Di-OA in rat plasma at three different concentrations (1.02, 5.10, and 10.2 μg/mL) were 100.17%, 97.46%, and 99.76%, and the mean absolute recoveries were 97.17%, 99.68%, and 96.36%, respectively.

The concentrations of Oxy-Di-OA did not markedly change during the freezing and thawing cycles. In addition, the spiked plasma samples were found sustainable at room temperature for 24 h. The concentrations of Oxy-Di-OA in the methanol solutions ranged from 97.46% to 100.17% of the initial concentration after storing at room temperature for 24 h. Therefore, these data indicated Oxy-Di-OA was stable in plasma biomatrix and the processed samples under such circumstances. Based on the results, the Oxy-Di-OA biosample preparation procedure can be achieved for its pharmacokinetic study.

#### 2.6.2. Pharmacokinetic Study and Data Analysis

**Figure 5:**
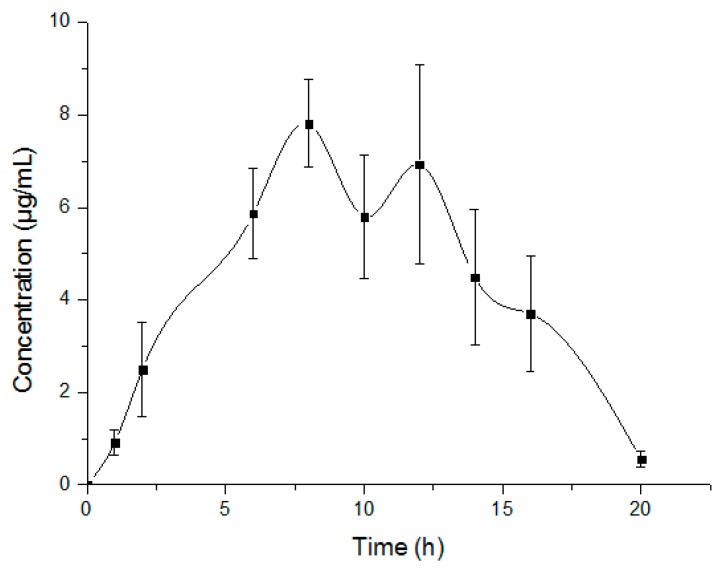
Plasma concentration-time profiles of Oxy-Di-OA after oral administration (mean ± SD, *n* = 6).

The developed method and the validated bioanalytical assay were implemented for the pharmacokinetic study of Oxy-Di-OA in rats. The mean plasma concentration versus time profile is represented in Figure 5 and the pharmacokinetic parameters are shown in Table 6. Oxy-Di-OA behaved according to the non-compartment model after single-dose oral administration in six rats. It was absorbed at a slow rate and reached the maximum plasma concentration (*C*_max_ = 8.18 ± 0.66 μg/mL) within 10 ± 2.19 h. The plasma concentration of Oxy-Di-OA attained an elimination half-life at 2.19 ± 0.71 h. The area under the concentration-time curve from *t* = 0 to the last time (AUC) (μg·h/mL) was 88.0194 ± 0.66 for Oxy-Di-OA.

## 3. Discussion

In the present study, we mainly investigated the protective effects of Oxy-Di-OA on CCl_4_-induced liver fibrosis in rats. It is generally accepted that CCl_4_ under laboratory conditions is one of the most common and widely used liver intoxicators today [18,19].

The histological results showed that the normal structure of lobules was destroyed and pseudolobules formed in the model group. Additionally, the increased serum ALT and AST also confirmed that the liver fibrosis was successfully developed in rats. In our CCl_4_-induced chronic liver injury model, we observed that the livers in Oxy-Di-OA with low- and high-dose treated rats displayed less injury in the histological assessment compared to those in the CCl_4_-only treated group. From Figure 2, compared with the bifendate group, the high-dose group showed less histological damage. Immunohistochemical staining is an important aspect to demonstrate the effects of Oxy-Di-OA on CCl_4_-induced liver fibrosis in rats. Numerous studies have demonstrated that liver fibrosis displayed an increased expression of TGF-β1. Furthermore, many studies have shown that the factor has a close relationship with the progress of liver fibrosis [20,21,22,23,24]. Thus, the levels of TGF-β1 in cells may reveal the degree of liver fibrosis. According to Figure 3, results suggested that Oxy-Di-OA in the high-dose group and the low-dose group can obviously reduce the level of TGF-β1. In particular, in the high-dose group, apoptosis proved that Oxy-Di-OA reduces the level of TGF-β1, further illustrating that Oxy-Di-OA can play a role in CCl_4_-induced liver fibrosis in rats. Hepatic fibrosis and damage caused by CCl_4_ were attenuated by Oxy-Di-OA administration. It is well accepted that CCl_4_ is metabolized by cytochrome p450 (CYP2E1 isoform) into the hepatotoxic radicals trichloromethyl CCl_3_• and Cl_3_COO• that covalently bind to cell constituents leading to chain lipid peroxidation. As a result, the situations of serious hepatic injury with marked elevations of serum aspartate aminotransferase (AST) and alanine aminotransferase (ALT) appeared after CCl_4_ intraperitoneal injection [25]. In the present study, both low and high doses of Oxy-Di-OA abrogated the CCl_4_ effects on plasma ALT and AST. This suggests that Oxy-Di-OA plays a role in ALT and AST release mechanisms. In addition, liver and spleen indices can also describe, secondarily, the effect of Oxy-Di-OA. After administration of CCl_4_, the rat’s liver and spleen indices apparently ascended. With the administration of bifendate and Oxy-Di-OA, those indices declined, conversely. Such a situation also agrees with former research [26]. This indicated that bifendate and Oxy-Di-OA have the same effect to relieve the symptoms of liver fibrosis in rats. Results of the acute toxicity test in vivo indicated that Oxy-Di-OA is a potent oral anti-hepatic fibrosis therapeutic with low toxicity.

On the other hand, a highly-validated, sensitive, and specific HPLC technique for the quantitation of Oxy-Di-OA was developed. The adequate selectivity, sensitivity, precision, and appropriate retention time make it suitable for high-throughput pharmacokinetic study. We developed a simple and reliable HPLC method to quantify Oxy-Di-OA in rat plasma. The analytical method was successfully applied to identify the pharmacokinetic profile of Oxy-Di-OA in rats. Furthermore, it was found that Oxy-Di-OA had a longer elimination half-life than its parent compound, oleanolic acid [27].

## 4. Materials and Methods

### 4.1. Animals and Housing Environment

Healthy Sprague-Dawley (SD) rats (weight: 180–220 g) of both sexes and male Kunming mice (weight: 18–22 g) were used in the experiments and were obtained from Vital River Laboratory Animal Technology Company Limited (animal production license permit no. SCXK-(A) 2006-0009). All animals were maintained under standard conditions with lights on from 7:00 to 19:00 at 25 ± 1 °C and a relative humidity of 50% ± 5%. The animals were used in experiments after two days of adaptive feed and were housed at the animal facility of the Pharmacological Animal Medicine Center, College of Basic Medicine, Beijing University of Chinese Medicine, Beijing, China. Principles of laboratory animal care were followed and all experiments were carried out in accordance with the “Regulation for the Administration of Affairs Concerning Experimental Animals” (State Council of China, 1988). And the Animal Care and Use Committee is the School of Chinese Pharmacy, Beijing University of Chinese Medicine (Date: 22 March 2015; No.: 201503-22).

### 4.2. Reagents

Both Oxy-Di-OA and internal standard (IS) (Figure 3A) were synthesized in our laboratory. The positive drug (bifendate) was purchased from Guangzhou Xingqun (Pharmaceutical) Co., Ltd. (Guangzhou, China). CCl_4_ was purchased from Beijing Chemical Reagent Factory (Beijing, China). Methanol of HPLC grade was purchased from Sigma Chemical (St. Louis, MO, USA). The TGF-β1 assay kit was obtained from Boster Biotech Co., Ltd. (Wuhan, China). All other chemicals, such as olive oil, were commercially available and were of analytical reagent (AR) grade. Analysis by HPLC was carried out on an Agilent 1100 series chromatographic system (Agilent Corporation, Santa Clara, CA, USA).

### 4.3. Experimental Procedure

After a two-week acclimation period, 52 rats were randomly divided into five groups (each group approximately consisted of ten rats). (1) Normal group: rats were fed with regular diet and distilled water and were injected intraperitoneally with olive oil (2 mL/kg body weight per day, i.p. twice per week) for nine weeks; (2) Model group: rats were feeding with the same quantity as the normal group and were injected with 40% CCl_4_/olive oil (2 mL/kg body weight, twice per week) for nine weeks to induce liver fibrosis; (3) Low-dose group: rats were fed with the same quantity as the normal group and were administered intraperitoneal injections with 40% CCl_4_/olive oil (2 mL/kg body weight, twice per week) and with a low dose (14 mg/kg body weight, once per day) Oxy-Di-OA treatment for nine weeks; (4) High-dose group: rats were fed with the same quantity as the normal group and were treated with 40% CCl_4_/olive oil (2 mL/kg body weight twice per week) and with a high dose (28 mg/kg body weight, once per day) Oxy-Di-OA treatment for nine weeks; (5) Positive control group: rats were fed with the same quantity as the normal group and were administered 40% CCl_4_/olive oil (2 mL/kg body weight, twice per week) and with bifendate (10.5 mg/kg body weight, once per day) for nine weeks.

At 48 h after the final injection of CCl_4_, all rats were anaesthetized with chloral hydrate, and the blood samples were collected from the aorta abdominalis. The serum samples were obtained by centrifugation at 3000 rpm for 15 min to obtain the supernatant and were kept frozen at −80 °C until biochemical analysis. Livers and spleens were immediately removed, rinsed in ice-cold saline, and weighed. The data were used for calculating liver and spleen indices. Then a portion of each liver was fixed in 4% buffered formaldehyde, embedded in paraffin blocks, and stained with hematoxylin-eosin (H&E) for histological and biochemical analyses. The rest of the tissues were stored at −80 °C until required.

The LC system is made up of an Agilent 1100 HPLC system (Agilent Corporation, USA) coupled with a reverse-phase Agilent TC-C18 analytical column (4.6 mm × 250 mm, 5 µm particle size). The column temperature set at 25 °C. The column used during the pharmacokinetic assay was eluted at a flow rate of 1 mL/min, and the solvent consisted of 98% methanol and 2% aqueous solution. The detection wavelength was 278 nm and the injection volume was 10 μL.

### 4.4. Histological Assay

The rats’ livers were carefully isolated and fixed with 4% buffered formaldehyde solution for 24 h. Then small fixed tissues were dehydrated and embedded in paraffin and then cut into 5 μm sections. Sections were stained with hematoxylin-eosin (H&E) to observe liver fibrosis with a light microscope. Stage assessment of hepatic fibrosis was rated on five levels [28,29,30]. Level 0, no fibrosis; level 1, mild fibrosis, fibrosis only occurring in the portal area; level 2, moderate fibrosis, peripheral fibrosis in the portal area; level 3, severe fibrosis, fibrous septum accompanied by intralobular structural disorders, but no hepatic cirrhosis; level 4, early hepatic cirrhosis; and level 5, cirrhosis.

### 4.5. Immunohistochemical Staining

Immunostaining was conducted as paraffin-embedded liver tissue sections of 5 µm thickness to determine the expression and distribution of TGF-β1 in the liver tissue of rats. Tissues were treated with 3% H_2_O_2_ in distilled water for 10 min to block endogenous peroxidase activity followed by another wash, and then blocked with 5% BSA blocking buffer for 20 min. Then, after endogenous peroxidase blockage, the sections were incubated with TGF-β1 primary antibody (1:50 dilution) at 4 °C overnight. The next day, they were washed three times with PBS (phosphate-buffered saline) and then incubated with HRP-labelled goat-anti-rabbit secondary antibodies at 37 °C for 30 min. After being washed with PBS three times, cells were treated with SABC (strept avidin-biotin complex) at 37 °C for 20 min. After that, PBS was used to wash again, then the samples were developed with a DAB (3,3′-diaminobenzidine) stain experiment. After observing the reaction with DAB chromogen, the samples were counterstained with hematoxylin and covered with a glycerin gel. The expression of TGF-β1 in liver tissues was analyzed under a light microscope (Olympus, Tokyo, Japan).

### 4.6. Measurement of Plasma Transaminase Activities

According to the corresponding manufacturer’s protocols, plasma samples were prepared after finishing animal experiments and determined using ALT and AST diagnostic kits. The ALT and AST enzyme activities were measured by the third affiliated hospital of Beijing University of Chinese Medicine (Beijing, China).

### 4.7. Liver and Spleen Indices

We used the percentage of the body weight to represent relative weights of the liver and spleen, respectively. The indices of liver and spleen were expressed as *W*_l_/*W*_m_ and *W*_s_/*W*_m_, respectively. The *W*_l_ and *W*_s_ represented the average liver and spleen weight of each group, respectively, and *W*_m_ was the average rat body weight of each group.

### 4.8. Acute Toxicity Assay

According to previous studies [31,32], Oxy-Di-OA was further investigated for its approximate LD_50_ and 95% confidence interval (CIs) value in mice. Male Kunming mice, weighing 20 ± 2 g were randomly divided into eleven groups of six individuals matched by weight and size. The mice had fasted for 12 h, but water was allowed ad libitum before the acute toxicity experiments.

#### 4.8.1. Acute Toxicity Assay via Intraperitoneal Injection

The maximum suspended dose (900 mg/kg) of Oxy-Di-OA was prepared in bean oil solution. One group of mice was injected intraperitoneally with bean oil as a normal control. Then the other five groups of mice were given intraperitoneal injections of Oxy-Di-OA in doses of 178, 422, 600, 750, and 900 mg/kg, respectively. The mice were observed continuously for 1 h after such treatment and then intermittently for 4 h, and thereafter over a period of 24 h. The mice were further observed for up to seven days following treatment for any situations of poisoning and deaths and the latency of death. Some abnormal behaviors were recorded.

The acute toxicity assay via intraperitoneal injection was evaluated by LD_50_ and 95% CIs which were calculated by Käber assessment.

#### 4.8.2. Acute Toxicity Assay via Gavage

One group of mice was orally administered bean oil as a normal control. Then the other four groups of mice were given gavage of Oxy-Di-OA in doses of 200, 500, 1000, and 2000 mg/kg, respectively. The mice were observed continuously for 1 h after such treatment and then intermittently for 4 h, and thereafter over a period of 24 h. The mice were further observed for up to seven days following treatment for any signs of toxicity, death, and the latency of death. Abnormal symptoms were recorded immediately.

The acute toxicity test via gavage was evaluated by LD_50_.

### 4.9. Pharmacokinetic Study

#### 4.9.1. Preparation of Standard Solution, Quality Control Working Solution, and Samples

Stock solution of Oxy-Di-OA (1.02 mg/mL) and quality control working solution of IS (50.4 μg/mL) were prepared by dissolving Oxy-Di-OA and IS in methanol. A series of Oxy-Di-OA standard working solutions at 0.255, 0.510, 1.020, 2.040, 5.10, 7.65, 10.20, 15.30, and 20.40 μg/mL were prepared, respectively, with methanol by further diluting the stock solution of Oxy-Di-OA. QC working solutions (1.02, 5.10, and 10.20 μg/mL) of Oxy-Di-OA were prepared in a like fashion. All of the solutions were stored in the refrigerator at 4 °C in the dark. The solution should be returned to room temperature before use. The working solutions of Oxy-Di-OA and of IS were stable for at least one month under the described conditions.

Healthy SD rats had no access to food until 12 h after dose administration, while water was free to access throughout the experiments. To determine the drug concentrations and to calculate the pharmacokinetic parameters, blood was obtained from the ophthalmic vein and collected with heparinized microcentrifuge tubes. The plasma was separated by centrifuging the blood samples at 4000 rpm for 15 min. Plasma stored in a freezer at −20 °C was thawed at 4 °C before treatment. After that, 15 μL of IS (50.4 μg/mL) working solution was added to such a plasma sample (200 μL), followed by vortex-mixing for 1 min to obtain the mixture. Then we added 600 μL acetonitrile to the mixture and vortexed for 5 min. After that, the mixture was centrifuged at 12,000 rpm for 5 min at 4 °C. The organic phase was quickly moved to another clean test tube and was evaporated to dryness by nitrogen at 45 °C. The residue was then reconstituted in 100 μL methanol, and stirred and vortexed for 1 min. The solution was centrifuged at 12,000 rpm for 5 min to obtain the supernatant. Finally, 10 μL of supernatant was injected into the HPLC system for further analysis.

#### 4.9.2. Method Validation

##### Calibration Curve and Linearity

The calibration curve of Oxy-Di-OA was prepared as follows: 200 μL blank rats’ plasma with 10 μL standard working solutions were mixed to achieve concentrations ranging from 0.255 to 20.4 μg/mL. Then 15 μL IS quality control working solution was added to each mixture, respectively. The linearity was evaluated with the linear correlation coefficient (*r*) of the calibration curve.

##### Precision, Limit of Detection, and Quantitation

Precision of an analytical method articulates how close the data values are to each other for a number of measurements acquired from numerous samplings of the identical homogeneous sample under the same analytical conditions and expressed as the intra-day and inter-day RSD [33]. Quality control samples (QC samples) were determined at low, middle, and high concentrations. They were prepared by blank plasma spiked with QC working solutions of Oxy-Di-OA, respectively. The values of intra-day and inter-day RSD were determined in the QC samples on the same day and on five sequential days, respectively. The limit of detection for Oxy-Di-OA was defined as the amount of drug at which the signal to noise ratio of the testing peak was 3:1. The limit of quantitation was the lowest concentration on the standard curve [34].

##### Extraction Recovery and Stability

The extraction recovery of Oxy-Di-OA from the QC samples included relative recovery and absolute recovery. Relative recovery was calculated from the theoretical concentration (*C*_t_) and the observed concentration (*C*_o_) of Oxy-Di-OA following to the equation: relative recovery (%) = (*C*_o_/*C*_t_) × 100%, which was also expressed as the accuracy [35]. Absolution recovery (%) = (*A*_o_/*A*_t_) × 100%. 

The stability of Oxy-Di-OA was investigated by analyzing the QC samples over three levels under different conditions, including −20 °C for 10 and 20 days (long-term stability), and room temperature for one day (short-term stability). In addition, the concentration of Oxy-Di-OA would be detected in the above samples to compare with the initial value in order to determine whether it has changed or not.

##### Pharmacokinetic Study and Data Analysis

Oxy-Di-OA was dissolved in normal saline before using. Before administration of drug, six SD rats had no access to food overnight but water was allowed free access. A single dose (500 mg/kg) of Oxy-Di-OA solution was administered orally to rats. After that, the blood samples were collected with heparinized microcentrifuge tubes at 1, 2, 6, 8, 10, 12, 14, 16 and 20 h. Plasma was separated by centrifugation (4000 rpm, 10 min, and 15 °C) and kept at −20 °C until necessary.

### 4.10. Statistical Analysis

Results were expressed as the mean ± S.D. Statistical comparisons between experimental groups was performed using one-way ANOVA followed by Student’s *t*-test. Statistical significance was achieved if *p* < 0.05. All statistical analysis was performed using SAS 9.2 software (available on: http://thefilesiwant.net/sas-free-download-t2767.html).

## 5. Conclusions

In this paper, we mainly performed the histological assessment, immunohistochemical staining, serum biochemistry, and liver and spleen indices to demonstrate the protective effects of Oxy-Di-OA on CCl_4_-induced liver fibrosis. Acute toxicity in vivo was carried out to evaluate the toxicity of Oxy-Di-OA. Finally, the performance of the pharmacokinetic study was used for the dynamic regulation of Oxy-Di-OA in rats. On the one hand, histological assessment and immunohistochemical staining intuitively exhibited the effect of anti-liver fibrosis. On the other hand, another two assessments displayed the effect of anti-hepatic fibrosis from processing and analyzing data. Altogether, the results show that Oxy-Di-OA has good performance against anti-hepatic fibrosis. Acute toxicity testing showed a low toxicity of Oxy-Di-OA. Pharmacokinetic study reveals the dynamic regulation of Oxy-Di-OA in vivo. In summary, based on the studies of pharmacology and pharmacokinetic evaluations, Oxy-Di-OA presents a valuable candidate for anti-hepatic fibrosis drug discovery and development.

## Figures and Tables

**Table 1:** Effects of Oxy-Di-OA on serum alanine aminotransferase (ALT) and aspartate aminotransferase (AST) (mean ± SD).

Groups	Dose (mg/kg)	*n*	ALT (U/L)	AST (U/L)
Normal	-	12	50.04 ± 11.59	130.80 ± 45.67
Model	-	12	99.78 ± 55.68 *	209.83 ± 111.08 *
Bifendate	10.5	9	54.54 ± 21.40 ^#^	122.56 ± 39.18 ^##^
Oxy-Di-OA	14	11	77.58 ± 39.58 *	122.00 ± 52.19 ^##^
Oxy-Di-OA	28	11	67.06 ± 51.59 ^#^	141.89 ± 71.57 ^#^

Data were expressed as mean ± SD; * significant difference from normal group at *p* < 0.05; ^#^, ^##^ significant difference from model group at *p* < 0.05 and *p* < 0.01, respective.

**Table 2:** Liver index and spleen indices in different experimental groups.

Groups	Dose (mg/kg)	*n*	Liver Index (%)	Spleen Index (%)
Normal	-	12	2.70 ± 0.18	0.17 ± 0.03
Model	-	12	3.72 ± 0.50 *	0.28 ± 0.09 *
Bifendate	10.5	9	3.57 ± 0.34 *	0.24 ± 0.04 *
Oxy-Di-OA	14	11	3.46 ± 0.38 *	0.24 ± 0.06 *
Oxy-Di-OA	28	11	3.15 ± 0.36 *^,#^	0.26 ± 0.05 *

Data were expressed as mean ± SD; * significant difference from control group at *p* < 0.05; ^#^ significant difference from model group at *p* < 0.05.

**Table 3:** Results of Oxy-Di-OA on mortality of the acute toxicity test via intraperitoneal injection in mice.

Group	Mice Number Start/End	Dose (mg/kg)	Death Rate (%)	LD50 (mg/kg)	95% CIs (mg/kg)
1	6/6	178	0	714.83	639.73–798.73
2	6/6	422	0		
3	6/6	600	0		
4	6/2	750	67		
5	6/1	900	83		

**Table 4:** Intra- and inter-day precision of Oxy-Di-OA in rat plasma (mean ± SD, *n* = 5).

Concentration Added (μg/mL)	Concentration Observed (μg/mL)		Precision (RSD %)	
	Intra-Day	Inter-Day	Intra-Day	Inter-Day
1.02	1.0156 ± 0.0246	1.0351 ± 0.0183	2.43	1.76
5.10	4.9083 ± 0.0839	4.7791 ± 0.2039	1.71	4.24
10.2	10.0974 ± 0.0548	9.7475 ± 0.3434	0.54	3.52

**Table 5:** Extraction recovery of Oxy-Di-OA in rat plasma (mean ± SD, *n* = 5).

Concentration Added (μg/mL)	Relative Recovery		Absolute Recovery	
	*C*_o_/*C*_t_ %	RSD %	*A*_o_/*A*_t_ %	RSD %
1.02	100.17 ± 0.90	0.90	97.17 ± 4.25	4.38
5.10	97.46 ± 0.76	0.78	99.68 ± 3.48	3.49
10.2	99.76 ± 1.06	1.07	96.36 ± 3.14	3.26

**Table 6:** Main pharmacokinetic parameters of Oxy-Di-OA in rats.

Pharmacokinetic Parameters	Units	Mean ± SD
AUC_last_	μg·h/mL	90.21 ± 15.68
CL	mL/h/kg	5575.84 ± 992.65
*V*_z_	mL/kg	18,290.17 ± 9453.59
*t*_1/2_	H	2.19 ± 0.71
*C*_max_	μg/mL	8.18 ± 0.66
*T*_max_	H	10.00 ± 2.19
MRT	H	9.48 ± 0.48
